# The impact of negative cognitive emotion regulation strategies on impulsive behaviors in middle school students: the mediating effect of cognitive fusion and experiential avoidance

**DOI:** 10.3389/fpsyt.2026.1782634

**Published:** 2026-08-24

**Authors:** Jingru Liu, Yanrong Wang

**Affiliations:** 1 School of Nursing, Ningxia Medical University, Yinchuan, Ningxia, China; 2 Mental Health Center, General Hospital of Ningxia Medical University, Yinchuan, Ningxia, China

**Keywords:** cognitive fusion, experiential avoidance, impulsive behaviors, mediating effect, negative cognitive emotion regulation strategies

## Abstract

**Background:**

Early adolescents are susceptible to impulsive behaviors due to immature cognitive control. Such behaviors are associated with elevated risks of disciplinary infractions, delinquent acts, and mental health disorders. Although cognitive emotion regulation strategies, cognitive fusion, and experiential avoidance have been identified as correlates of impulsive behavior, their integrated interrelationships remain underexplored.

**Objective:**

To investigate the effects of negative cognitive-emotional regulation strategies on impulsive behaviors among middle school students, and to examine the chained mediating roles of cognitive fusion and experiential avoidance in this relationship.

**Methods:**

A total of 756 middle school students completed self-report questionnaires, including the Cognitive-Emotional Regulation Questionnaire, the Barratt Impulsiveness Scale, the Cognitive Fusion Questionnaire, and the Acceptance and Action Questionnaire.

**Results:**

(1) Negative cognitive-emotional regulation strategies were significantly positively correlated with impulsive behaviors, cognitive fusion, and experiential avoidance. Cognitive fusion and experiential avoidance were both significantly positively correlated with impulsive behaviors. (2) Both cognitive fusion and experiential avoidance exhibited significant effects in the individual and chained mediation of the relationship between negative cognitive-emotional regulation strategies and impulsive behaviors.

**Conclusion:**

Improving negative cognitive-emotional regulation strategies and reducing levels of cognitive fusion and experiential avoidance may be important avenues for reducing impulsive behaviors among middle school students. These findings provide empirical guidance for educators and mental health professionals in conducting mental health education and implementing interventions for impulsive behaviors among adolescents.

## 1 Introduction

Impulsive behavior is defined as actions that are poorly conceived and are often risky and inappropriate (1). Adolescence is an important stage of individual physical and mental development, but also a high incidence of behavior problems (2). Neuroimaging evidence demonstrates that adolescence is marked by neurodevelopmental asynchrony between the prefrontal cortex, which subserves executive control and deliberative decision-making, and the limbic system, which mediates emotional processing and reward valuation (3). This maturational asynchrony, coupled with pubertal changes in dopaminergic signaling that amplify reward sensitivity, contributes to heightened impulsivity and emotional reactivity during this developmental period (4). Such neurocognitive vulnerabilities increase the likelihood of externalizing behavioral difficulties, including truancy, school absenteeism, and delinquent conduct. If not corrected in time, serious problems such as substance abuse and bullying in schools may occur (5). Empirical studies have shown that adolescent impulsive behavior is closely related to antisocial behavior, high-risk sexual behavior, and delinquency (6). In addition, some scholars have found that individuals with high impulsivity are prone to mental illnesses or psychological disorders, such as emotional outbursts, emotional eating, panic disorder, and self-inflicted suicidality (7–10). Therefore, it is of great significance to explore the influencing factors and mechanism of impulsive behaviors in middle school students.

Relevant research has found that the selection of cognitive-emotional regulation strategies plays a crucial role in impulsive behavior (11). Cognitive-emotional regulation strategies are defined as a series of psychological mechanisms through which individuals manage and regulate their emotional responses by actively adjusting and restructuring their cognitive processes when faced with emotionally arousing events (12). Based on Garnefski’s Cognitive Emotional Regulation (CER) model, these strategies can be categorized into positive cognitive-emotional regulation strategies (such as acceptance, positive refocusing, rational analysis, positive reappraisal, and refocusing plans) and negative cognitive-emotional regulation strategies (including self-blame, blaming others, catastrophizing, and rumination), both of which have distinctly different effects on an individual’s psychological adaptation and behavioral regulation (13). Among these, the core characteristic of negative cognitive-emotional regulation strategies lies in trapping individuals in a vicious cycle of failed emotional regulation by maintaining or even amplifying the intensity and duration of negative emotional experiences. Specifically, when individuals rely on negative strategies such as rumination, catastrophic thinking, or self-blame over the long term, their emotional regulation processes will exhibit systemic dysfunction: negative emotions continuously accumulate and consume a significant number of cognitive resources, leading to a significant weakening of the prefrontal cortex’s inhibitory function over impulsive behavior, thereby reducing behavioral control. Existing research has confirmed a close association between negative cognitive-emotional regulation strategies and impulsive behavior (14–16). A recent systematic review suggests that impulsive behavior can be regarded as a constituent element of cognitive-emotional regulation disorders in patients with obsessive-compulsive disorder (17). Frequent use of negative cognitive-emotional regulation strategies is often accompanied by a higher tendency toward impulsive responses (18). However, previous studies have rarely specifically examined the relationship between negative cognitive-emotional regulation strategies and impulsive behavior in secondary school students, and there is a lack of in-depth exploration of the underlying mechanisms. Therefore, based on the CER theoretical model, this study aims to systematically explore the impact of negative cognitive-emotional regulation strategies on impulsive behavior in secondary school students and further elucidate the mediating mechanisms involved.

Within the core pathological mechanisms of Acceptance and Commitment Therapy (ACT), cognitive fusion and experiential avoidance are two key constructs; both are frequently regarded as critical mediating variables in the mechanisms underlying adolescent mental health and problem behaviors (19). For adolescents who frequently employ negative cognitive-emotional regulation strategies, they are fixated on defending their conceptualized self. Following negative events, they become trapped in negative thoughts, finding it difficult to distinguish inappropriate self-perceptions from reality, thereby leading to cognitive fusion and amplifying their experience of distress (20). Although experiential avoidance provides individuals with a short-term, highly effective pathway to emotional relief, according to the thought suppression paradox, such avoidance strategies often prove counterproductive: the more deeply an individual avoids distress, the more likely their emotional regulation functions are to become trapped in a negative cycle, with psychological distress and behavioral problems consequently intensifying (21, 22). It can therefore be hypothesized that negative cognitive-emotional regulation strategies may contribute to functional dysregulation of the emotional regulation system through psychological rigidity mechanisms characterized by cognitive fusion and experiential avoidance. This impairment may hinder effective processing of negative emotions, ultimately manifesting as impulsive behavior.

In summary, based on the theoretical framework of cognitive-emotional regulation, this study systematically examines the relationship between negative cognitive-emotional regulation strategies, cognitive fusion, experiential avoidance, and impulsive behaviors among secondary school students. It aims to elucidate the underlying mechanisms of impulsive behaviors, with the goal of providing empirical evidence for the early prevention and scientific intervention of impulsive behaviors in adolescents. This study proposes the following hypotheses: (i) Negative cognitive-emotional regulation strategies significantly influence impulsive behaviors in secondary school students; (ii) Cognitive fusion and experiential avoidance serve as sequential mediators between negative cognitive-emotional regulation strategies and impulsive behaviors.

## 2 Methods

### 2.1 Participants and the procedure

This study utilized convenience sampling to recruit students from four public middle schools in Yinchuan, Ningxia. School selection followed a structured procedure: researchers initially contacted eight schools and ultimately selected four mainstream public institutions matched on school size, teaching quality, and student demographic composition. Special education schools were explicitly excluded to minimize sample bias. Inclusion Criteria: Participants must be between 12 and 16 years of age and provide informed consent to participate voluntarily. Exclusion Criteria: Participants who, based on medical history and clinical examination, have physical conditions such as hearing or vision impairments that prevent them from completing the questionnaire, or those diagnosed with a mental disorder by a psychiatric hospital or with a relevant medical history.

Prior to any data collection activities, official written approval was obtained from the administrative departments of all four participating schools. The survey was administered on-site by researchers who had undergone standardized training. Given that all participants were minors, ethical guidelines for research involving human subjects were strictly followed. Standardized written informed consent forms were distributed to both the students and their parents or legal guardians. The consent form clearly and comprehensively explained the study’s purpose, research procedures, data collection methods, the absolute anonymity of all responses, strict data confidentiality, and the participants’ right to withdraw from the study at any time without any negative consequences. Only students who provided written assent themselves and whose parents or legal guardians had signed the written informed consent form were included in the final sample. After receiving standardized instructions, participants completed the questionnaire independently in a private space to ensure anonymity and confidentiality. Questionnaires were collected on-site, and researchers verified the completeness of responses immediately to ensure data quality.

### 2.2 Measurements

#### 2.2.1 Cognitive emotion regulation questionnaire

The Cognitive Emotion Regulation Questionnaire (CERQ) is a 36-item instrument designed to assess cognitive emotion regulation strategies. The Chinese version of the CERQ was translated and validated by Zhu, and it has demonstrated good psychometric properties in Chinese adolescents (23). Confirmatory factor analysis supported the original nine-factor structure (CFI = 0.981, RMSEA = 0.062) and indicated good measurement invariance across gender and time. This study analyzed four dimensions: self-blame, contemplation, catastrophizing, and blaming others. A 5-point scale was used, with higher scores indicating a tendency for subjects to use a particular strategy when faced with an event. The Cronbach’s α coefficient of the CERQ in this study was 0.94.

#### 2.2.2 Barratt impulse scale

The Barratt Impulsivity Scale (BIS) is a well-established instrument for measuring trait impulsivity. This study employed the Chinese version of the BIS (24). This version was developed through a rigorous process of translation and cultural adaptation, including independent forward-backward translation and expert panel review for cultural appropriateness, to ensure its applicability within the Chinese context. Substantial prior evidence supports its sound psychometric properties and widespread use with Chinese adolescent samples. This 30-item scale comprises three dimensions: motor impulsivity, attentional impulsivity, and non-planning impulsivity. Items are rated on a 4-point Likert scale, with higher total scores indicating higher levels of impulsive behavior and trait impulsivity. In the present study, the BIS demonstrated good internal consistency, with a Cronbach’s α coefficient of 0.81.

#### 2.2.3 Cognitive integration scale

The Cognitive Integration Scale (CIS) is a unidimensional instrument designed to assess cognitive integration and psychological rigidity. The present study employed the Chinese version of the CIS (25). Confirmatory factor analysis supported the one-factor model (CFI = 0.99, RMSEA = 0.06). The Chinese CIS has demonstrated robust construct validity and internal consistency in prior research and has been successfully used in studies with Chinese adolescent populations. This 9-item scale uses a 7-point Likert format, with higher total scores indicating higher levels of cognitive integration and psychological rigidity. In this study, the CIS exhibited excellent internal consistency, with a Cronbach’s α coefficient of 0.95 in our sample of adolescents.

#### 2.2.4 Acceptance and action questionnaire-second edition

This study employed the Chinese version of the AAQ-II, which was originally developed by Bond and subsequently validated by Cao using a standard translation-back-translation procedure with a sample of Chinese students (26). This version demonstrates high internal consistency (α = 0.88), test-retest reliability (r = 0.80), and construct validity (CFI = 0.99, RMSEA = 0.06). The instrument consists of 7 items rated on a 7-point Likert scale, with higher total scores indicating greater levels of experiential avoidance and psychological rigidity. In the present study, the Cronbach’s α coefficient for the AAQ-II was 0.93.

### 2.3 Statistical analyses

IBM SPSS 25.0 was used for data cleaning, management, and statistical analysis. First, normality tests and outlier identification were conducted; confirmed outliers were verified, and sensitivity analyses were performed. Continuous variables were described as means ± standard deviations (M ± SD), and categorical variables were presented as frequencies (percentages). Pearson correlation analysis was used to examine the relationships among the variables. The PROCESS macro program developed by Hayes (Model 6) was employed to test the chained mediating effects of cognitive fusion and experiential avoidance on the relationship between negative cognitive-emotional regulation strategies and impulsive behavior. The 95% confidence interval for the indirect effects was estimated using the bootstrap method (5,000 repeated samples). If the confidence interval did not include zero, the mediating effect was considered statistically significant.

## 3 Results

### 3.1 Descriptive statistics

A total of 796 questionnaires were distributed in this study, with 756 valid questionnaires collected, yielding an effective response rate of 95%. Among these, 19 were excluded due to participants withdrawing midway or parental requests for withdrawal, while 31 were excluded because of missing responses to at least one questionnaire item. The sample comprised 366 boys (48%) and 390 girls (52%), with a mean age of 13.83 years (SD = 1.44). (See **Table 1** for details).

**Table 1:** Demographic characteristics.

Variables	Categories	Participants(N=756)
Age, *M ± SD*		13.83 ± 1.44
Gender, n (%)		
	Male	366(48%)
	Female	390(52%)
Ethnicity, n (%)		
	Han Chinese	383(51%)
	Other	373(49%)
Parental Educational Attainment, n (%)		
	Junior high school or below	400(53%)
	Above junior high school	356(47%)
Parental Health, n (%)		
	Good	520(69%)
	Chronic diseases	236(31%)
Parental Marital Status, n (%)		
	Harmony	651(86%)
	Single parent/remarriage	105(14%)

(Table showing demographic characteristics of 756 adolescent participants, including age, gender, ethnicity, parental education, health, and marital status).

In this study, Q-Q plot analysis indicated that the distribution of scores for negative cognitive emotion regulation strategies, cognitive fusion, experiential avoidance, and impulsive behavior approximated a normal distribution, with absolute skewness and kurtosis values below 3. aA multicollinearity test was performed for all variables, revealing a maximum variance inflation factor of 1.29, suggesting no significant multicollinearity issues.

### 3.2 Common method bias test

In light of the fact that the data from all participants in the present study were gathered through self-rating scales, there is a possibility of common method bias. Therefore, the Harman single factor method was employed to examine this issue (27). Following the primary analysis, three distinct factors were identified. The findings revealed that the first common factor explained 35.72% of the variance, and the crucial criterion was below 40%, suggesting the absence of common method bias in this study.

### 3.3 Correlation analysis

A Pearson correlation analysis of the variables revealed that negative cognitive emotion regulation strategies were positively correlated with cognitive fusion (r = 0.28, p < 0.01), experiential avoidance (r = 0.26, p < 0.01), and impulsive behavior (r = 0.26, p < 0.01), indicating that the more frequently middle school students employ negative cognitive-emotional regulation strategies, the higher their levels of cognitive fusion, experiential avoidance, and impulsive behavior. Cognitive fusion was significantly positively correlated with experiential avoidance (r = 0.45, p < 0.01) and impulsive behavior (r = 0.31, p < 0.01), and experiential avoidance was also significantly positively correlated with impulsive behavior (r = 0.34, p < 0.01). (See **Table 2** for details).

**Table 2:** Means, standard deviations, and correlations among variables.

Variables	*M ± SD*	1	2	3	4
1. CERQ	44.60 ± 11.53	1			
2. CIS	26.77 ± 12.70	0.28**	1		
3. AAQ	18.36 ± 10.05	0.26**	0.45**	1	
4. BIS	66.03 ± 14.79	0.26**	0.31**	0.34**	1

(CERQ, cognitive emotion regulation strategies; CIS, cognitive integration scale; AAQ, The Acceptance and Action Scale; BIS, The Barratt Impulsivity Scale; *p<0.05, **p<0.01, same below. **Table 2**. Means, standard deviations and correlations among CERQ, CIS, AAQ and BIS).

### 3.4 Analysis of intermediation effects

**Figure 1:**
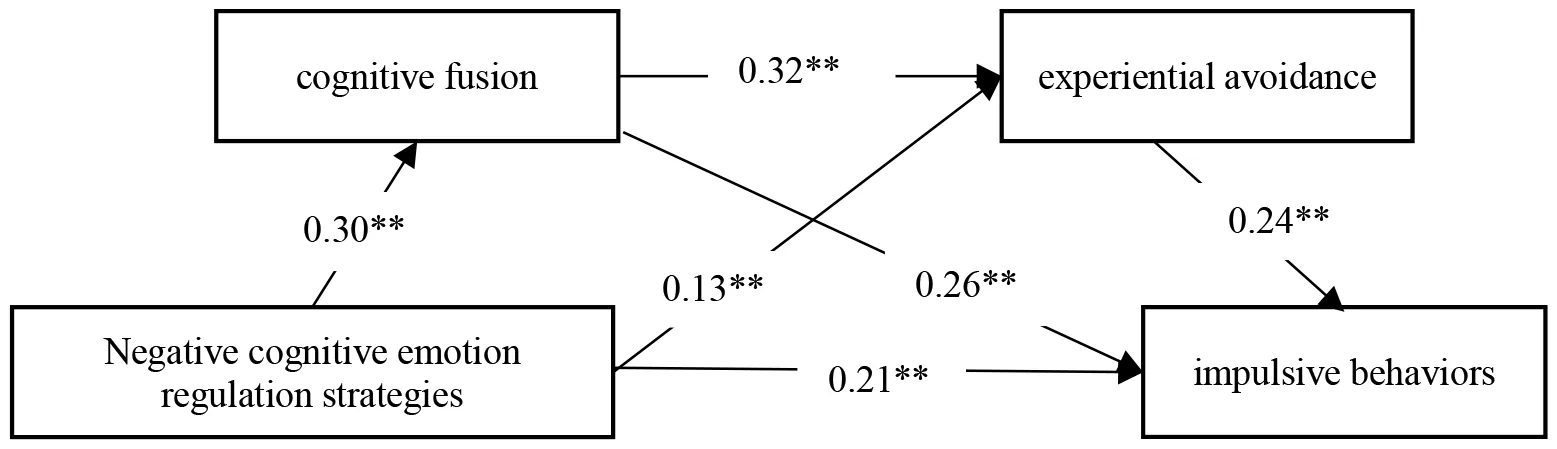
Sequential mediation model of negative cognitive emotion regulation strategies on impulsive behaviors: the roles of cognitive fusion and experiential avoidance.

We employed PROCESS Model 6 to examine the mediating effects of cognitive fusion and experiential avoidance on the relationship between negative cognitive-emotional regulation strategies and impulsive behavior, with results presented in **Table 3** and **Figure 1**. The results indicate that, after controlling for gender and age, negative cognitive emotion regulation strategies positively predict cognitive fusion (β = 0.30, 95% CI [0.23, 0.38]), experiential avoidance (β = 0.13, 95% CI [-0.07, -0.19]), and impulsive behavior (β = 0.21, 95% CI [0.12, 0.29]). Cognitive integration positively predicted experiential avoidance (β = 0.32, 95% CI [0.27, 0.37[) and impulsive behavior (β = 0.26, 95% CI [0.17, 0.34]). Experiential avoidance positively predicted impulsive behavior (β = 0.24, 95% CI [0.14, 0.35]). The study found that the indirect effect of negative cognitive-emotional regulation strategies on impulsive behavior through cognitive integration was significant (β = 0.08, 95% CI [0.04, 0.11]). The mediating effect (CERQ → CIS → BIS) accounted for 23.5% of the total effect and represented the core pathway explaining the influence of negative cognitive-emotional regulation strategies on impulsive behavior. Furthermore, experiential avoidance mediated the relationship between negative cognitive-emotional regulation strategies and impulsive behavior (β = 0.03, 95% CI [0.01, 0.06]). The mediating effect (CERQ → AAQ → BIS) accounted for 8.8% of the total effect, indicating a significantly weaker strength. This difference supports the theoretical hypothesis that experiential avoidance primarily functions as a downstream outcome of cognitive fusion rather than as an independent primary driver of impulsive behavior. Further findings confirmed that negative cognitive-emotional regulation strategies exert an indirect effect on impulsive behavior through cognitive fusion and experiential avoidance (B = 0.02, 95% CI [0.01, 0.04]). The mediating effect (CERQ → CIS → AAQ → BIS) accounted for 5.9% of the total effect. Although the effect size was small, this chain effect represents a unique mechanistic pathway that cannot be reduced to the sum of individual mediating effects. It suggests that cognitive integration creates cognitive conditions that necessitate experiential avoidance, which in turn depletes self-regulatory resources and manifests as impulsive behavior.

**Table 3:** Bootstrap mediating effects of negative cognitive emotion regulation strategies and impulsive behavior.

Paths	*B*	Estimate	Bootstrap 95% *CI*	
			LLCI	ULCI
CERQ →CIS →BIS	0.08	23.5%	0.04	0.11
CERQ →AAQ→BIS	0.03	8.8%	0.01	0.06
CERQ →CIS →AAQ→BIS	0.02	5.9%	0.01	0.04
Indirect effect	0.13	38.2%	0.09	0.18
Direct effect	0.21	61.8%		
Total effect	0.34	100%		

(Bootstrap mediation effects showing path estimates, confidence intervals, and effect proportions for CERQ, CIS, AAQ, and BIS.).

To examine potential heterogeneity in the serial mediation mechanism across distinct negative cognitive emotion regulation strategies, we conducted separate serial mediation analyses (Hayes’s PROCESS Model 6) for self-blame, rumination, catastrophizing, and blaming others, respectively; results are presented in **Table 4**. The results indicate that, after controlling for gender and age, the total indirect effect was strongest for self-blame (B = 0.37, 95% CI [0.22, 0.52]), followed by rumination (B = 0.35, 95% CI [0.23, 0.50]) and catastrophizing (B = 0.31, 95% CI [0.19, 0.44]), whereas blaming others yielded the weakest total indirect effect (B = 0.26, 95% CI [0.15, 0.39]). Importantly, for self-blame, the single-mediator pathway via experiential avoidance failed to reach statistical significance (B = 0.05, 95% CI [−0.01, 0.12]). Unlike the other strategies, catastrophizing demonstrated the strongest single-mediator effect through experiential avoidance (B = 0.10, 95% CI [0.04, 0.17]).

**Table 4:** Standardized indirect effects of different negative cognitive emotion regulation strategies on impulsive behavior.

Predictor	Total indirect effect	Indirect effect 1	Indirect effect 2	Indirect effect 3
Self-blame	B=0.37, 95%CI [0.22, 0.52]	B=0.23, 95%CI [0.13, 0.35]	B=0.05, 95%CI [-0.01, 0.12]	B=0.08, 95%CI [0.04, 0.14]
Rumination	B=0.35, 95%CI [0.23, 0.50]	B=0.21, 95%CI [0.11, 0.32]	B=0.07, 95%CI [0.02, 0.14]	B=0.07, 95%CI [0.03, 0.11]
Catastrophizing	B=0.31, 95%CI [0.19, 0.44]	B=0.16, 95%CI [0.08, 0.26]	B=0.10, 95%CI [0.04, 0.17]	B=0.05, 95%CI [0.02, 0.09]
Blaming others	B=0.26, 95%CI [0.15, 0.39]	B=0.14, 95%CI [0.06, 0.23]	B=0.07, 95%CI [0.02, 0.14]	B=0.05, 95%CI [0.02, 0.08]

(**Table 4** shows standardized indirect effects of four negative cognitive emotion regulation strategies on impulsive behavior via three pathways: cognitive fusion (CIS) alone, experiential avoidance (AAQ) alone, and a serial chain (CIS→AAQ). Self-blame and rumination showed the strongest total indirect effects. Catastrophizing had the strongest single-mediator effect through AAQ. Blaming others showed the weakest effects across all pathways. Indirect effect 1: CERQ →CIS →BIS; Indirect effect 2: CERQ →AAQ→BIS; Indirect effect 3: CERQ →CIS →AAQ→BIS).

## 4 Discussion

This study found that negative cognitive emotion regulation strategies predict impulsive behavior in middle school students, consistent with previous research (15), which indicates that individuals who tend to use negative cognitive emotion regulation strategies are more likely to exhibit impulsive behavior. Hypothesis (i) was thus confirmed. Research indicates that cognitive-emotional regulation strategies are closely associated with impulsive behavior. Individuals who employ negative cognitive-emotional regulation strategies often experience more psychological problems and are prone to expressing anger, impulsivity, and acting out (28). Cognitive-emotional regulation training can reduce impulsive behavior in individuals with post-traumatic stress disorder (29). Studies have shown that impulsive behavior among college students is significantly associated with negative cognitions and negative coping strategies (30).

The results of this study indicate that cognitive fusion and experiential avoidance serve as sequential mediators in the relationship between negative cognitive emotion regulation strategies and impulsive behavior, thereby confirming Hypothesis (ii). Specifically, the more frequently individuals employ negative cognitive-emotional regulation strategies, the higher their levels of cognitive fusion and experiential avoidance, and consequently, the more impulsive behavior they exhibit. Individuals who habitually employ negative cognitive-emotional regulation strategies tend to engage in automatic negative rumination when exposed to negative stimuli or potentially negative information, impairing their ability to effectively regulate negative cognitions. During this process, individuals often conflate subjective, one-sided cognitions and negative emotional experiences with objective reality, becoming persistently preoccupied with thoughts related to negative events, ultimately resulting in cognitive fusion (31). As a core dimension of psychological inflexibility, cognitive fusion further predisposes individuals to adopt an experiential avoidance coping style. To escape the persistent negative emotional experiences and psychological distress caused by cognitive fusion, individuals frequently employ rigid avoidance strategies—restricting their cognitive processing scope and avoiding negative internal experiences and external situations in an attempt to alleviate immediate psychological discomfort. However, while experiential avoidance may provide temporary emotional relief, it ultimately reinforces the vicious cycle of negative cognitive-emotional states, exacerbates psychological inflexibility, and leads to behaviors incongruent with personal goals (32). When an individual’s behavioral patterns persistently diverge from their value orientations, psychological conflicts and negative emotional experiences in daily life intensify. Existing research indicates that individuals with higher levels of cognitive fusion and experiential avoidance often experience more severe emotional distress and exhibit poorer cognitive-emotional regulation abilities. Deficits in these core psychological functions further impair their capacity to inhibit impulsive behavior, significantly increasing the likelihood of such behavior (33). This mechanism has been further validated in individuals with chronic non-cancer pain (CNCP). Relevant studies demonstrate that CNCP patients, who are chronically affected by pain and entrenched in persistent negative emotions, are more likely to adopt negative cognitive-emotional regulation strategies. These individuals struggle to redirect their attention from the pain experience and its adverse consequences to other meaningful aspects of life, making them highly susceptible to an avoidance pattern centered on pain evasion. Consequently, they engage in impulsive behaviors that prioritize immediate avoidance needs over long-term goals, consistent with the core findings of this study (34). Thus, cognitive fusion and experiential avoidance may be key mechanisms through which cognitive-emotional regulation strategies influence impulsive behavior.

The analysis revealed substantial heterogeneity among the four negative cognitive emotion regulation strategies in both the magnitude of their mediating effects and the specific pathways through which they operated. Self-blame and rumination emerged as the primary drivers of the serial mediation chain, accounting for the largest total indirect effects on impulsive behavior. From the perspective of Relational Frame Theory (RFT), both self-blame and rumination represent self-referential forms of repetitive negative thinking that progressively strengthen rigid, derived relational networks linking negative events, adverse self-evaluations, and aversive emotional states. This persistent relational responding intensifies the fusion between self-concept and negative internal experiences (35), thereby explaining why the single-mediator pathway via cognitive fusion consistently accounted for the largest proportion of the total effect for both strategies. Notably, however, the two strategies diverged in their downstream mechanisms. For self-blame, the independent mediating effect of experiential avoidance did not reach statistical significance. Previous studies have shown that self-blame compromises impulse control primarily by directly intensifying cognitive fusion and triggering acute cognitive-affective overload, rather than by initially activating deliberate experiential avoidance (36). In contrast, rumination not only consolidated cognitive fusion but also progressively accumulated unresolved emotional tension. This escalating tension, in turn, motivated experiential avoidance, thereby completing the full serial mediation pathway (37).

From the perspective of developmental psychology, the behavioral patterns of self-blame and rumination are highly consistent with the core developmental characteristics of early adolescence. Due to asynchronous brain development, the regulatory function of the prefrontal cortex remains immature, while the limbic system continues to dominate self-related cognitive processes (38). Middle school students at this developmental stage display heightened self-centeredness and are also highly sensitive to external evaluations and academic feedback (39). When encountering setbacks, adolescents in this age group tend to attribute negative outcomes to internal factors, thereby entering a cycle of self-criticism and repetitive thinking. These developmental dynamics amplify the pathogenic potential of self-focused cognitive strategies, accelerating progression toward cognitive fusion and subsequent impulse dysregulation (40).

Catastrophizing exhibited a distinct pathway profile. Although its total indirect effect and serial mediation effect were relatively modest, the single-mediator pathway through experiential avoidance was the strongest for catastrophizing across all four strategies, which is consistent with previous research findings (41). Catastrophizing magnifies the perceived threat and aversiveness of events through exaggerated relational framing, rendering internal affective experiences increasingly intolerable and directly motivating experiential avoidance as an escape strategy (42). This pattern is consistent with the heightened reactivity of the adolescent amygdala to threat cues and the still-developing capacity of the prefrontal cortex to downregulate catastrophic appraisals (43). Nevertheless, the overall effect of catastrophizing remained weaker than that of self-blame and rumination because it centers on the negative consequences of events rather than on self-devaluation, thereby exerting a relatively weaker influence on the core self-related cognitive fusion network.

Blaming others exhibited the weakest total indirect effect and the smallest serial mediation effect, which is consistent with previous research findings (18). As an externally directed cognitive regulation strategy, blaming others involves attributing the cause of adverse events to external factors rather than to the self. This external attribution pattern forms relational networks centered on external stimuli, which penetrate less deeply into the self-concept system and result in a lower degree of cognitive fusion. Consequently, its subsequent impacts on experiential avoidance and impulsive behavior are comparatively limited self-focused limited. Self-focused negative cognitive strategies exert more detrimental effects on adolescents’ internal cognitive-affective regulation than externally directed strategies (44).

In summary, this study investigated the psychological mechanisms underlying the relationship between negative cognitive emotion regulation strategies and impulsive behavior, with a particular focus on the serial mediating effects of cognitive fusion and experiential avoidance. The results demonstrate that negative cognitive emotion regulation strategies can directly influence impulsive behavior levels in middle school students, as well as indirectly influence impulsive behavior through a serial mediation process wherein cognitive fusion is first heightened, followed by an increase in experiential avoidance. The total proportion of the mediating effect reached 38.2%. These findings suggest that reducing the use of negative emotion regulation strategies while enhancing psychological detachment and acceptance may decrease impulsive behavior, which has important implications for mental health interventions among adolescents. Schools, families, and society should collectively address adolescents’ cognitive-emotion regulation strategies and levels of psychological inflexibility. Educators can guide students through mental health curricula to identify and reduce the use of negative cognitive strategies. Rather than implementing generalized emotion regulation training, school-based prevention programs can adopt differentiated intervention schemes. By implementing approaches such as mindfulness training and cognitive defusion to reduce cognitive fusion and decrease experiential avoidance of negative emotions, these interventions may enhance psychological flexibility and emotion regulation skills, thereby strengthening students’ psychological coping capacities when facing adverse events. School counselors and clinical psychologists could also develop structured preventive intervention programs based on this serial mediation model, integrating cognitive strategy training with psychological flexibility development to more effectively mitigate the risk of impulsive behavior among middle school students.

## 5 Limitations and future directions

This study has several limitations that should be carefully considered when interpreting the results. First, although the sample exhibits some heterogeneity in terms of ethnic composition and family socioeconomic background, the findings are difficult to generalize to remote areas, private or special education schools, and clinical adolescent populations; caution is also warranted when generalizing across regions and cultures. Second, the study employed a cross-sectional design, which can only reveal associations and predictive relationships between variables but cannot establish causal relationships. Third, all variable data were derived from self-report questionnaires. Although bias was controlled through procedures such as anonymous administration and reverse scoring, inherent limitations—such as social desirability bias and response omissions—could not be completely eliminated and may have had a potential impact on the results.

Future research could be expanded in the following areas. First, longitudinal tracking or intervention study designs could be employed to clarify the dynamic causal relationships among variables. Second, the sampling strategy could be refined by utilizing stratified random sampling to enhance sample representativeness, incorporating diverse geographic regions, school types, and clinical populations to examine the model’s robustness across different groups. Third, data collection methods should be diversified through multi-source assessment approaches to mitigate self-report bias. Fourth, digital contexts continuously shape adolescents’ affective experiences and reward processing, which may moderate the strength of the pathways identified in this study. Unfortunately, indicators of digital behaviors were not included in the current investigation. Future research could integrate measurements of social media engagement and problematic smartphone use into the model to examine whether digital behaviors amplify or buffer the serial mediation chain from negative cognitive emotion regulation strategies to impulsive behavior.

## 6 Conclusion

This study elucidates the underlying mechanisms through which negative cognitive-emotional regulation strategies influence impulsive behavior in middle school students via the serial mediation of cognitive fusion and experiential avoidance, thereby offering a novel theoretical perspective for mental health education and intervention targeting adolescent impulsive behavior.

## Data Availability

The raw data supporting the conclusions of this article will be made available by the authors, without undue reservation.
